# Soluble biomarkers for Neuromyelitis Optica Spectrum Disorders: a mini review

**DOI:** 10.3389/fneur.2024.1415535

**Published:** 2024-05-16

**Authors:** Rachel E. Rodin, Tanuja Chitnis

**Affiliations:** ^1^Department of Neurology, Brigham MS Center, Brigham and Women’s Hospital, Boston, MA, United States; ^2^Harvard Medical School, Boston, MA, United States

**Keywords:** NMO, neurofilament, glial fibrillary acidic protein, neutrophil, lymphocyte

## Abstract

The Neuromyelitis Optica Spectrum Disorders (NMOSD) constitute a spectrum of rare autoimmune diseases of the central nervous system characterized by episodes of transverse myelitis, optic neuritis, and other demyelinating attacks. Previously thought to be a subtype of multiple sclerosis, NMOSD is now known to be a distinct disease with unique pathophysiology, clinical course, and treatment options. Although there have been significant recent advances in the diagnosis and treatment of NMOSD, the field still lacks clinically validated biomarkers that can be used to stratify disease severity, monitor disease activity, and inform treatment decisions. Here we review many emerging NMOSD biomarkers including markers of cellular damage, neutrophil-to-lymphocyte ratio, complement, and cytokines, with a focus on how each biomarker can potentially be used for initial diagnosis, relapse surveillance, disability prediction, and treatment monitoring.

## Introduction

Neuromyelitis Optica Spectrum Disorders occur in 1–10 per 100,000 people worldwide across ages and ethnicities, with females and people of Asian or African ancestry being most often affected (1). The vast majority of NMOSD patients have a relapsing disease course and accumulate disability over time due to incomplete recovery after recurrent attacks (2). Despite recent breakthroughs in treatment options for NMOSD, there are no established guidelines on whom to treat with immunosuppressive medications and for how long, as relapses can occur even after prolonged periods of remission (3). There is a critical need for clinically validated biomarkers that can be used to guide care and ultimately improve the lives of NMOSD patients.

Based on 2015 international consensus diagnostic criteria, patients can be diagnosed with NMOSD if they have a positive AQP4-IgG test, at least one core clinical characteristic (including optic neuritis, acute myelitis, area postrema syndrome, acute brainstem syndrome, or symptomatic cerebral or diencephalic MRI lesions), and reasonable exclusion of alternative diagnoses (4). Over 80% of NMOSD patients fulfill these criteria with positive AQP4-IgG serology (5). A smaller fraction of NMOSD patients have AQP4-IgG-negative disease, and some of these patients are instead found to have anti-MOG antibodies. For purposes of this review, we will discuss biomarkers of AQP4-IgG-seropositive and seronegative NMOSD excluding cases with positive anti-MOG serology, now known as MOG-associated disease (MOGAD).

An ideal disease biomarker is sensitive and specific, easily sampled via non-invasive techniques, and affordable to assay. Valuable roles for biomarkers include aiding in diagnostic accuracy, predicting relapse, monitoring treatment response, and assessing safety of treatment discontinuation. Though strongly involved in the pathophysiology of NMOSD, AQP4-IgG is not a useful biomarker for monitoring disease activity or treatment response. Laboratories employ different methods of detecting AQP4-IgG and though a live cell-based assay is the gold standard, sensitivity and specificity vary considerably across other assays, leading to difficulty in standardizing the interpretation of titer results (6). While some studies have shown a strong correlation between serum AQP4-IgG titer and disease activity (7, 8), others have failed to replicate these findings (9–11). Some patients relapse despite negative seroconversion (12), whereas others do not relapse despite very high AQP4-IgG titers (10). For this reason, monitoring of serum AQP4-IgG has not been adopted as a standard practice in NMOSD patient care, and the field must instead turn to other biomarkers.

## Markers of cellular damage

Glial fibrillary acidic protein (GFAP) is an intermediate filament protein found in the cytoskeletons of astrocytes (13) and neurofilament light chain (NfL) is a scaffolding protein located primarily within neuronal axons (14). Since NMOSD is predominately an astrocytopathy with secondary neuronal damage, both GFAP and NfL are released into CSF due to cellular damage and can then be detected in serum, with serum levels tightly correlated to CSF levels (15). GFAP and NfL have emerged as promising biomarkers in many neurologic diseases including NMOSD.

### GFAP and NfL as diagnostic biomarkers

Serum GFAP (sGFAP) and serum NfL (sNfL) are elevated at baseline in NMOSD compared to healthy controls, and sGFAP is higher in AQP4-IgG-positive NMOSD when compared to both multiple sclerosis (MS) and to a lesser extent MOGAD (15–17), implying that sGFAP, but not sNfL, may assist in distinguishing NMOSD from other autoimmune CNS diseases. Notably, sGFAP does not appear to be as elevated in AQP4-seronegative NMOSD (18, 19), suggesting that the subset of patients with clinical NMOSD who test negative for AQP4-IgG may have a unique disease pathology which does not involve a primary astrocytopathy. Conversely, sNfL is elevated in both seropositive and seronegative NMOSD along with other demyelinating conditions, supporting a common role for secondary neuronal damage in all of these diseases (19).

Although a majority of studies find sGFAP to be significantly higher in AQP4-IgG-positive NMOSD compared to MS, there is overlap in the concentrations reported, and therefore no validated numeric sGFAP cutoff has been universally established to support NMOSD diagnosis. The ratio of sGFAP/sNfL is especially promising for differentiating seropositive NMOSD from MOGAD and MS (15, 16, 20), with a sGFAP/sNfL quotient above 5.71 being 73% sensitive and 75.8% specific for AQP4-IgG-positive NMOSD compared to MS (15). Importantly, the timing of serum sampling relative to most recent relapse must be carefully considered when interpreting cellular damage biomarkers. Both sNfL and sGFAP can remain elevated in remitted NMOSD compared to healthy controls, but sGFAP declines after relapse more quickly and to a greater extent than sNfL, which can remain quite elevated in NMOSD patients for many months to years after relapse (15, 16, 21), suggesting ongoing secondary neuronal damage long after the acute attack.

### GFAP and NfL as attack biomarkers

Another possible role for biomarkers of cellular damage is in monitoring for NMOSD attacks and distinguishing between true attacks vs. pseudo-attacks, or worsening symptoms in the absence of new MRI-visible disease activity, especially since a serum test would be timelier and more cost-efficient than repeated MRIs. In general, AQP4-IgG-positive NMOSD patients with higher baseline sGFAP have a shorter interval to the next attack compared to NMOSD patients with normal sGFAP (hazard ratio 3–11 within 0.5–2 years) (17, 22). The sGFAP level may then increase between 4-20x above an individual’s recent baseline at the time of an attack or within <1 week preceding an attack (15, 17, 21, 23, 24). Moreover, higher peak sGFAP levels correlate with attack severity, suggesting that more extensive astrocytic damage underlies more disabling disease (17). Beyond acute attacks, robust evidence has shown that higher sGFAP correlates with higher clinical disability scores in NMOSD (15, 16, 20, 22, 24), though it is unclear if this is due to ongoing immune-mediated astrocyte damage vs. chronic astrogliosis (25). Even in clinically stable patients, higher sGFAP correlates with retinal neuraxonal loss and worsening afferent visual function, suggesting a greater degree of subclinical chronic optic nerve damage (26).

While some studies have shown significant elevations in sNfL during NMOSD relapse compared to remission (24, 27), most evidence suggests that sNfL is not a useful biomarker for predicting relapse (15, 16, 22, 27). However, sNfL may be the strongest cellular damage biomarker for predicting accumulated disability (27). Other markers of CNS cellular damage including neuron-specific ubiquitin C-terminal hydrolase L1 (UCHL1), tau, neurofilament heavy chain (NfH), and astrocytic protein S100B have also been studied in NMOSD, though to a much lesser extent than GFAP and NfL. CSF S100B is elevated during acute attacks of NMOSD compared to MS and other neurologic diseases, is significantly higher in AQP4-IgG-positive NMOSD compared to seronegative disease, and correlates with Expanded Disability Status Scale (EDSS) during both attack and remission (28). CSF S100B also correlates with the number of spinal segments involved in acute myelitis associated with NMOSD, but is generally less predictive than CSF GFAP (29). Serum and CSF levels of S100B appear to be closely correlated (30), though insufficient studies have been conducted on serum S100B as a biomarker in NMOSD. CSF NfH is elevated in NMOSD compared to MS, but does not correlate with attack severity or disability (31). Serum levels of tau and UCHL1 are also elevated during attacks and are higher in AQP4-IgG-positive patients than in seronegative NMOSD (19), but do not predict relapse as well as sGFAP and do not predict disability as well as sNfL (27).

### GFAP and NfL in treatment response

In addition to aiding in diagnosis and attack monitoring, markers of cellular damage have a potential role in assessing response to treatment. Treatment with anti-CD20 agent rituximab has been shown to either stabilize (20) or decrease sGFAP over time (24) when compared to other immunosuppressive agents. Similarly, treatment with anti-IL-6 agent tocilizumab leads to slightly decreased levels of sGFAP over a period of 12 months when compared to prednisolone (24), and treatment with anti-CD19 drug inebilizumab results in significantly decreased sGFAP levels compared to placebo in as little as 12 weeks (17). Interestingly, in those patients who did experience clinical attacks while on inebilizumab there was no concomitant increase in sGFAP (17). This may indicate that the symptoms experienced by those patients were in fact pseudo-relapses without new disease activity, or that the biomarker profile of attacks is altered by anti-CD19 therapy. Most studies also show a significant decline in sNfL with immunosuppressive treatments (24, 27, 32). Additional research is needed to define sGFAP cutoff values that stratify relapse risk in NMOSD patients and to better understand the utility of monitoring GFAP, NfL and other cellular injury biomarkers in patients on immunosuppressive therapies.

## Neutrophil-to-lymphocyte ratio

Histopathologic samples of NMOSD lesions have abundant neutrophils, in contrast to MS lesions which have more macrophages and T-lymphocytes, and the CSF neutrophil count is elevated in approximately 60% of NMOSD patients during acute attacks (33). The neutrophil-to-lymphocyte ratio (NLR) in blood has emerged as a biomarker in numerous diseases including NMOSD (34). Though NLR is elevated in many inflammatory diseases, a small study including 15 MOGAD patients and 28 NMOSD patients showed that NLR > 2.86 is 75% sensitive and 86.7% specific for a diagnosis of NMOSD rather than MOGAD in patients who present with similar initial symptoms (35). Another study of 50 patients suggested that NLR >2.04 is 73% sensitive and 70.8% specific for diagnosing NMOSD over MS and MOGAD (36).

Results are mixed regarding the utility of NLR for stratifying NMOSD severity. One adult study found that at the time of hospitalization with first presentation of NMOSD, elevated NLR above 2.54 is an independent predictor of higher initial disability, though with a small odds ratio of 1.08 (37). NLR also predicts EDSS at 6 and 12 months post-attack in pediatric patients (38). Additionally, at the time of admission with first attack, NLR >2.38 may be up to 81.8% sensitive and 64.7% specific for upcoming relapse over an average follow-up time of 44 months, and NLR > 2.63 is up to 76.3% sensitive and 68% specific for poor functional recovery from the initial attack (39). Meanwhile, other studies have shown that while NLR is indeed elevated in NMOSD during both relapse and remission, it is not an independent predictor of outcome (40).

Larger studies will be necessary to confirm whether NLR is a reliable biomarker for distinguishing NMOSD from disease mimics and to determine if and when NLR rises prior to attacks. More work is also needed to determine how NLR is affected by treatments, apart from corticosteroids which cause increased NLR via neutrophil demargination. At present, data suggest that a very high NLR in a patient meeting diagnostic criteria for NMOSD may prompt consideration of aggressive treatment options for a presumably more severe form of the disease.

## Complement

The activation of complement has an established role in NMOSD pathophysiology (41, 42) and provides a promising target for drug discovery, with C5 cleavage inhibitor eculizumab showing excellent clinical efficacy in preventing NMOSD relapse (43). Multiple studies have shown that serum levels of C3 and C4 are lower in NMOSD during acute attacks when compared to controls and patients with MOGAD (44, 45). However, in clinically stable patients C3 and C4 trends are inconsistent, with one study showing lower C4 but not C3 (46), another showing lower C3 but not C4 (47), and another showing no difference in C3 or C4 when comparing remitted NMOSD patients to controls (48). Notably, these studies were performed on small cohorts and inconsistent results may be attributable to differences in assay techniques, duration of remission, and disease-modifying therapies. In a cohort of mixed relapse and remission states serum C1-inhibitor and C5 were both elevated in NMOSD compared to controls (49), whereas in a separate cohort of remitted patients there was no difference between NMOSD and controls in either of these biomarkers (47). Data on complement activation products are similarly inconsistent, with varying reports suggesting that serum sC5b-9 may be higher during relapse (50, 51), equivalent during relapse (52), or lower during remission (47) when compared to controls. In CSF samples sC5b-9, C5a, C10-inhibitor, and C1q are all elevated in NMOSD patients, though not all complement biomarkers can differentiate NMOSD from MS (53–55).

Multiple reports have shown correlations between components of the complement pathway and NMOSD disease severity. Higher serum C3a predicts a higher EDSS for a given attack, with elevated levels of C3a and elevated C3a:C3 ratio in patients who have relapsed within the past 6 months (50), despite other studies showing overall low C3 during active disease. Serum sC5b-9 is also significantly elevated during relapse compared to remission (52), and CSF sC5b-9 correlates with higher EDSS (54). Though CSF C5a is not significantly different between relapsing NMOSD and relapsing MS, in patients with NMOSD it does significantly correlate with the number of enhancing lesions seen on MRI (53).

To date only one small study of 3 NMOSD patients has examined the effect of treatment on complement, showing that eculizumab leads to a reduction in serum CH50 but no change in C3 or C4 (56). More studies with uniform metrics including remission vs. relapse states, CSF vs. serum testing, and treated vs. untreated patients will be needed in order to establish complement proteins as reliable disease activity biomarkers.

## Cytokines, chemokines, and T-lymphocytes

Many cellular and humoral immune mediators are important in NMOSD pathogenesis and are now being studied as potential biomarkers. IL-6 plays several roles in NMOSD, including promoting the survival of plasmablasts that secrete AQP4-IgG, and has proven to be an important drug target with the success of both tocilizumab and satralizumab (57). IL-6 is significantly increased in the CSF of NMOSD patients during initial attack compared to controls (58, 59) and in relapse compared to remission (60), and can be used to distinguish NMOSD from MS (61). CSF IL-6 also correlates with EDSS (62). The role of serum IL-6 as a biomarker is less clear. Some studies have identified elevated serum IL-6 in relapsing NMOSD patients compared to remission and controls (63), while others have failed to show a significant difference between NMOSD and controls (59, 64). Other reports suggest that serum IL-6 significantly correlates with risk of relapse, disease activity and brain atrophy (63, 65). More work is needed to determine whether serum IL-6 can be used as a biomarker of disease activity and to characterize the effect of treatment on both serum and CSF IL-6.

Beyond IL-6, there is also evidence for elevation of other TH17-related cytokines in NMOSD CSF including IL-17A, IL-8, IL-13, TGF-β1, IL-10, BAFF, and APRIL (59, 60, 66, 67). Serum data is less consistent, with significant elevations in serum TGF-β1 and IL-10 in AQP4-IgG-positive NMOSD patients compared to other groups, but no elevation in serum IL-17A (59). In terms of chemokines, CXCL13 is elevated in both CSF (68) and serum (69) during NMOSD attacks, reliably distinguishes NMOSD from MS, and correlates with EDSS (68, 69). Meanwhile, CSF elevations in CXCL1, CXCL5, and CXCL7 can distinguish NMOSD from MS, but do not correlate with disease severity (70). Serum CXCL5 is lower in NMOSD patients than in controls, but does not predict severity nor differentiate from MS (71).

Though NMOSD was initially thought to be mediated primarily by B cells, a prominent role of T cells has emerged. Granzyme B-expressing CD8+ T cells are markedly elevated in blood of NMOSD patients both in relapse and recovery when compared to controls, and can also be used to predict response to immunotherapy, with higher proportions of granzyme B expression correlating with inadequate clinical response to azathioprine, mycophenolate, or rituximab (72). CD4 + CXCR5 + PD-1 + T follicular helper cells are also elevated during NMOSD relapse and predict disability (69). T cell subsets are an intriguing potential drug target and biomarker that merit attention in future research.

## Other biomarkers

In addition to the large classes of biomarkers previously discussed, several other NMOSD biomarkers have been proposed. Adhesion molecules, which play a key role in breakdown of the blood–brain barrier leading to migration of peripheral immune cells and antibodies into CSF, are dysregulated in NMOSD. Specifically, elevated serum ICAM-2 and decreased serum PECAM-1 differentiate NMOSD from MS, and PECAM-1 negatively predicts EDSS (73). Neopterin, a nonspecific marker of cellular immune activation, is elevated in the CSF of patients with NMOSD compared to MS, and also predicts current relapse as opposed to remission with greater accuracy than other standard CSF tests including protein, cell count, and IgG index (74).

AQP4 is abundant in the medullary segment of renal collecting ducts, and though overt renal failure is uncommon in patients with NMOSD, markers of renal function may correlate with disease status. During acute attacks urine pH is significantly higher and urine specific gravity is lower in NMOSD compared to MS (75). Additionally, elevated GFR is an independent predictor of relapse in patients with AQP4-IgG-positive NMOSD (76). Due to ease of access, urine biomarkers of relapse and treatment response should be further investigated.

## Conclusion

**Figure 1:**
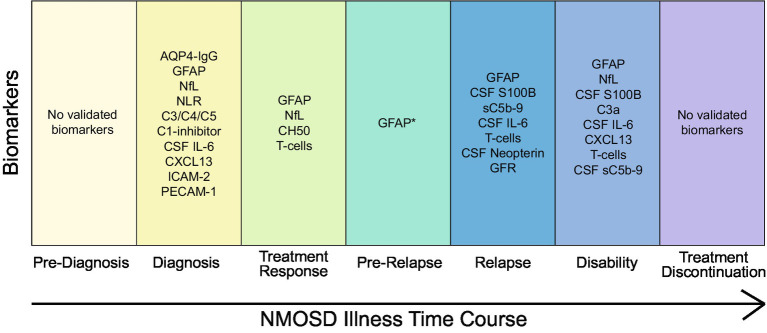
Summary of key NMOSD biomarkers and their clinical utility during various stages of disease. Except where indicated, all referenced biomarkers are from serum. *Relapse prediction for serum GFAP has only been shown within <1 week preceding relapse.

There are many promising biomarkers that have the potential to meaningfully improve the care of NMOSD patients throughout the course of the disease (Figure 1). Although many of the biomarker results discussed here are encouraging, much work remains to be done before these biomarkers will be widely accepted into clinical practice. Some biomarkers have been studied using older detection techniques including immunoblot and ELISA, whereas more recent studies have used newer technologies such as single-molecule arrays (SIMOA) (77), and it is not clear how results will translate across detection methods. Furthermore, as treatment options for NMOSD expand, studies will need to carefully consider the effects of different treatments on biomarker interpretation.

Nearly all statistically significant findings discussed in this review reflect comparisons between groups of patients, often with substantial overlap in numeric biomarker values. With the possible exception of sGFAP as a relapse biomarker when compared to an individual’s own baseline, no other biomarker is yet robust enough to be used at the individual patient level. One potential way to increase biomarker precision is by using z-scores. Particularly for NfL and GFAP, recent studies on other diseases have found that the use of z-scores, rather than raw biomarker values, can correct for variability within populations and enhance the determination of pathologic biomarker cutoff values (78, 79). Another possible way to increase the clinical applicability of NMOSD biomarkers could be via a combined disease severity or relapse risk score calculated from multiple biomarkers, which could have higher sensitivity and specificity compared to any single biomarker.

## Author contributions

RR: Writing – review & editing, Writing – original draft. TC: Writing – review & editing, Writing – original draft.
